# Natural Fiber Reinforced Composite Material for Product Design: A Short Review

**DOI:** 10.3390/polym13121917

**Published:** 2021-06-09

**Authors:** M. A. Azman, M. R. M. Asyraf, A. Khalina, Michal Petrů, C. M. Ruzaidi, S. M. Sapuan, W. B. Wan Nik, M. R. Ishak, R. A. Ilyas, M. J. Suriani

**Affiliations:** 1 Faculty of Ocean Engineering Technology and Informatics, Universiti Malaysia Terengganu, Kuala Nerus 21030, Terengganu, Malaysia; asyrafazman23@yahoo.com (M.A.A.); ruzaidi@umt.edu.my (C.M.R.); niksani@umt.edu.my (W.B.W.N.); 2 Department of Aerospace Engineering, Faculty of Engineering, Universiti Putra Malaysia, Serdang 43400, Selangor, Malaysia; asyrafriz96@gmail.com (M.R.M.A.); mohdridzwan@upm.edu.my (M.R.I.); 3 Laboratory of Biocomposite Technology, Institute of Tropical Forestry and Forest Products (INTROP), Universiti Putra Malaysia, Serdang 43400, Selangor, Malaysia; khalina@upm.edu.my (A.K.); sapuan@upm.edu.my (S.M.S.); 4 Faculty of Mechanical Engineering, Technical University of Liberec, Studentská 2, 461 17 Liberec, Czech Republic; michal.petru@tul.cz; 5 Marine Materials Research Group, Faculty of Ocean Engineering Technology and Informatics, Universiti Malaysia Terengganu, Kuala Nerus 21030, Terengganu, Malaysia; 6 Advanced Engineering Materials and Composites Research Centre (AEMC), Department of Mechanical and Manufacturing Engineering, Faculty of Engineering, Universiti Putra Malaysia, Serdang 43400, Selangor, Malaysia; 7 School of Chemical and Energy Engineering, Faculty of Engineering, Universiti Teknologi Malaysia, Johor Bahru 81310, Johor, Malaysia; 8 Centre for Advanced Composite Materials (CACM), Universiti Teknologi Malaysia, Johor Bahru 81310, Johor, Malaysia

**Keywords:** natural fiber composite, product design, sustainability design, design process

## Abstract

Natural fibers have attracted great attention from industrial players and researchers for the exploitation of polymer composites because of their “greener” nature and contribution to sustainable practice. Various industries have shifted toward sustainable technology in order to improve the balance between the environment and social and economic concerns. This manuscript aims to provide a brief review of the development of the foremost natural fiber-reinforced polymer composite (NFRPC) product designs and their applications. The first part of the manuscript presents a summary of the background of various natural fibers and their composites in the context of engineering applications. The behaviors of NFPCs vary with fiber type, source, and structure. Several drawbacks of NFPCs, e.g., higher water absorption rate, inferior fire resistance, and lower mechanical properties, have limited their applications. This has necessitated the development of good practice in systematic engineering design in order to attain optimized NRPC products. Product design and manufacturing engineering need to move in a mutually considerate manner in order to produce successful natural fiber-based composite material products. The design process involves concept design, material selection, and finally, the manufacturing of the design. Numerous products have been commercialized using natural fibers, e.g., sports equipment, musical instruments, and electronic products. In the end, this review provides a guideline for the product design process based on natural fibers, which subsequently leads to a sustainable design.

## 1. Introduction

A new product begins with an idea and ends with the physical production of the product. According to Milton and Rodgers [1], to minimize or reduce the impact of a product on the environment, it is necessary to reconsider its impact throughout its life cycle, such as how it is produced, the development process, usage, packaging, preservation, and recycling or disposal. When a product ignores environmental factors in its design process, designers are likely to face backlash from their customers. The competition of the product market nowadays is increasing; therefore, designers should consider the selection of environmentally friendly materials as the main criteria in their design.

Natural fiber composites are environmentally friendly materials that have attracted attention in the field of product manufacturing engineering [2,3]. Starting 3000 years ago, straw-reinforced clay was the first composite material to be used by the ancient Egyptians in their building construction. Research and development have proven that natural fibers have been successfully applied as reinforcements in the composites industry, such as for transportation, interior components, building, aircraft, and construction [4,5,6,7,8]. Furthermore, natural fiber composites have the advantages of being cheaper than synthetic composites, bio-degradable, abundantly available, renewable, and lightweight [9,10,11,12,13,14,15,16]. Natural fibers originate from three sources, namely, plants, animals, and minerals. There are more than 2000 types of fiber plants in the world, and these are mostly composed of cellulose, e.g., kenaf, sugar palm, bamboo, corn, cotton, flax, hay (from grass cutting), hemp, henequen, jute, pineapple leaf, banana, ramie, and sisal [17,18]. The use of natural fibers in composites can also solve some other problems, such as moderate energy consumption during production, leaving almost no carbon footprint, and reducing disposal problems [19,20,21,22].

Design is the first step in the manufacturing process; at this stage, many important decisions need to be made that will affect the result of a product. Therefore, several things need to be considered, such as manufacturing, assembly, cost, sales, maintenance, disposal, and recycling, early on in the design process. In addition, 70% of product manufacturing costs are determined at an early stage of the design process [23]. Product design using natural fiber composite uses the same method as other product design processes [24,25,26]. Product designers will determine the formal qualities of products manufactured by industry by focusing on aptness in function, use, ease of production, materials, cost, and the number of constituent parts. Additionally, they also concentrate on user experience—the interaction between users and products, types of meanings products evoke, and what sorts of emotions the products elicit [17]. Marzuki [27] also reported that designers need three things, namely, material, machinery, and method of manufacturing. This means that designers do not only have to produce quality designs, but they are also responsible for proposing and determining the appropriate materials so that the product can be produced at an affordable cost. This shows that the use of materials, human factors, and design are interrelated and can serve as a guideline to designers.

Fundamentally, design and manufacturing need to move in an integrated manner to complete the design process, including design concepts, material selection, and manufacturing process selection. Each problem needs to be addressed according to its respective expertise. Some experts will focus on the materials to be used, while others will focus on design concepts [24,28]. Design concepts and materials can be combined in a computer system, namely, computer-aided drawing (CAD) and finite element analysis (FEA).

## 2. Natural Fiber Reinforced Composite Material

### Natural Fiber

Natural fiber materials have become increasingly popular in the manufacturing industry and have been studied by many researchers. Natural fibers are divided into three categories, namely, cellulose-based, protein-based, and mineral-based, as shown in Table 1. Natural fibers are sustainable materials that are available in nature and have advantages as listed in Table 2. The compositions of natural fibers can be divided into three main components, which are cellulose, hemicellulose, and lignin. Table 3 displays the chemical composition of the natural fibers, in which the chemical composition and cell structures are quite complex and differ between plant parts and origins. Depending on the cellulose crystallinity, the physical, chemical, and mechanical behaviors of the lignocellulosic fibers vary from one to another [29,30]. Generally, natural fiber’s main constituent is cellulose, at 30–80%, followed by hemicellulose at 7–40%, and 3–33% lignin, as shown in Table 3.

## 3. Composites

In 1980, a fiberglass-reinforced plastic composite (GFRP) known as fiberglass and carbon fiber-reinforced polymer composite (CFRP) was designed. Composites are formed from a combination of two or more materials of physical and chemical difference [41]. This combination consists of the reinforcement phase, in the form of fibers, pieces, or particles [67,68], being embedded in other materials, referred to as the matrix phase [69,70,71]. Reinforcement is load-bearing, while the matrix phase serves as a binder of the reinforcing material and distributes the load between the fibers. According to Elanchezhian et al. [9], the matrix also acts as a material to protect the fiber material from damage, before, during, and after composite processing. This combination is able to produce new materials with better properties than the individual material [15,72].

On the other hand, composite hybrids involve a combination of two or more fibers in a matrix [10]. Composite hybrids also have broad prospects in product design as manufacturing materials. Hybrid composites can overcome several natural fiber deficiencies, e.g., low mechanical properties, high absorption properties, poor adhesion, and poor thermal stability during the process [4]. The results of the studies by Rashid et al. [73] on Kevlar reinforced with woven coir found that the impact strength showed minimal enhancement while the breakable properties of pure epoxy composites were decreased. According to Jawaid et al. [74], the addition of fibers and coupling agents significantly improved the thermal stability (e.g., decomposition and residue content) of the hybrids. In addition, a study conducted by Masoodi and Pillai [75] found that hybrid jute composites possessed high resistance to water absorption. However, the strength of hybrid jute composites was decreased as the humidity was increased. To reduce this effect, more jute fiber fractions are needed. Past studies have also found that the combination of natural fiber with synthetic fiber should be preferably recommended. For instance, a study on long kenaf fibers with Kevlar highlighted the effectiveness of materials, as well as cost material savings. The result of the study showed that reinforcing 20% of Kevlar’s weight within the composite kenaf enabled it to absorb a maximum energy of 12.76 J [10]. This has proven that the combination of Kevlar fiber with a kenaf composite is capable of improving energy absorption and imparting higher strength properties.

## 4. Product Design for Natural Fiber Composite (NFC)

Industrial design (ID) is the professional practice of designing products, devices, objects, and services used by millions of people around the world every day [76]. Product design is one of the sub-areas in industrial design that include medical and safety equipment and home appliances. A good product design needs to go through a long process, namely, the design process, before entering the manufacturing stage. According to Abidin et al. [77], furniture design has a very wide scope and comprises furniture in houses, offices, and public places. For transportation design, it consists of land, sea, and air vehicles, e.g., cars, motorcycles, buses, sea trucks, ships, jet skis, helicopters, and airplanes. According to Ramani et al. [78], in 2007, the industrial sector in the United States of America had produced over 1235 × 106 metric tons of carbon dioxide gas that would further complicate the restoration of the greenhouse gases.

### 4.1. Selection Material in Product Design

**Figure 1:**
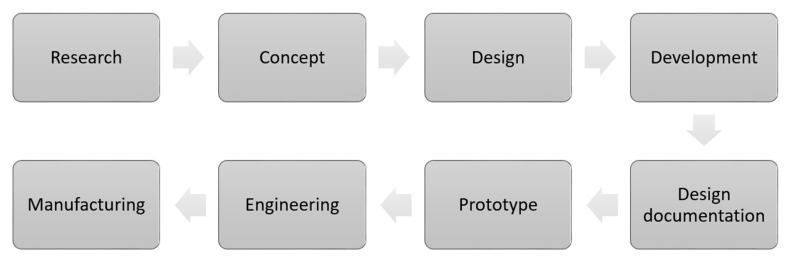
Design process flow.

Product designers can use natural fiber composite materials in design proposals in order for the design to be promoted as an eco-design. Eco-design is also known as design for the environment, and is defined as the process of “integrating a systematic environmental system into product design and development” [79]. Designers need to be more careful in choosing the right natural fiber for a product. According to research conducted by Karana [80], the choice of material often depends on the material that has been used before, to ensure that the material to be used is safe. However, this method causes the selection of materials to be limited. The selection of material plays an important role in the production of an innovative product. According to a study by Taekema and Karana [39], materials can be distinguished according to their sensory properties, e.g., blurred texture and transparency, or mechanical properties such as tensile strength, thermal conductivity, and the ability of materials to be processed and shaped, for example, they can be painted or injected. This process needs to be completed in the conceptual design phase. Figure 1 shows the design process starting from the initial stage of idea sketching to 2D rendering [81]. Therefore, as an introduction, designers should form an overview of these key properties, e.g., sensorial properties (such as its velvet-like texture and its transparency), technical properties (such as it specific tensile strength), and formability properties (such as its ability to undergo injection molding or being paintable) to inspire and stimulate them to decide on a particular material. Unless technical requirements are defined at the outset of the project, product designers consider the technical properties at an overview level, and not in detail at the conceptual design stage [39]. According to Elvin Karana et al. [82], designers can use the Meanings of Material (MoM) model as a guideline for material selection. These guidelines can also be applied by product designers to select natural fibers that are appropriate for the designed product. In addition, natural fibers also have intangible properties, such as their relation to trends and value to the culture, and emotions evoked by a material. These circumstances play important roles in helping product designers to make decisions in material selection.

### 4.2. Evaluation Concept for Product Design

Product designers need reliable, rigorous, and robust methods for evaluating and selecting their design proposals. Choosing the right method is very important, and choosing the wrong product design proposal to be developed can be very costly to the manufacturer in terms of money, time, and other valuable resources. Designers need to constantly evaluate the direction of their design concept while, at the same time, creating many concepts to choose from. When considering the selection of proposals, product design specifications (PDS) are very useful because they serve as evaluation parameters in the process [1]. Poor PDS is also one of the reasons for low-quality or unsuccessful products. Specifications are desired as measurable parameters of features that facilitate the realization of a function [83]. Table 4 shows some of the key points that should appear in a PDS. These are taken from a pilot study conducted by Azman et al. [84] into redeveloping a face mask design for hajj people.

Matrix evaluation, or the Paugh method, is a quantitative technique used by designers to evaluate their proposed design concepts by ranking them against the set criteria stated in the PDS [1]. This method was invented by a British engineering design professor Stuart Paugh, who was considered a pioneer in product design development, and this method has been used worldwide in the field of design for manufacturing [23]. The selection of concept design proposals is one of the processes involved in narrowing down a number of alternative proposals and aims to select one for further development and refinement. Below is the decision matrix model (Paugh’s method). As stated by Mahmud et al. [83], a significant increase in the information available on product design specifications (PDS) during the design process leads to a lower rate of product desertion. The process of selecting the most satisfactory design proposal according to the PDS is very important in ensuring that the proposed design concept does not deviate from the guidelines as stated in the PDS. Matrix evaluation can help designers, engineers, manufacturers, marketing staff, users, clients, and buyers to reduce ambiguity and confusion in the evaluation and selection process, resulting in clearer communication and the delivery of successful new products to market more frequently.

### 4.3. Development of Product Design by Integrating Design for Sustainability with Other Concurrent Engineering Techniques

**Figure 2:**
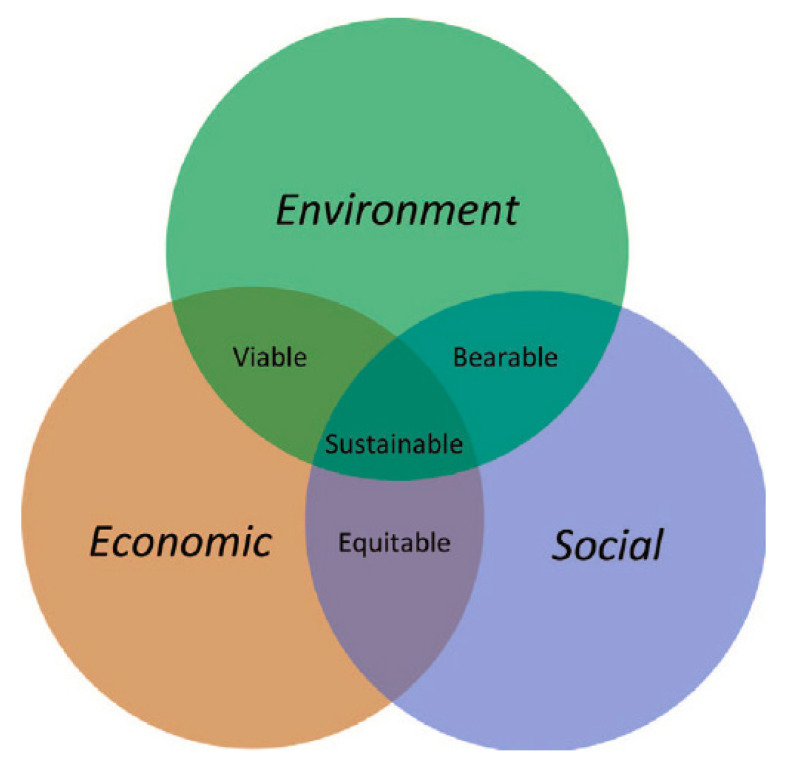
The pillars of sustainability [88].

During the process of the development of NFCs products, product engineers have to implement the concept of design for sustainability (DfS) in order to promote sustainable products. Design for sustainability (DfS) could play a vital role in directing us towards sustainable consumption and production, which is defined based on the four pillars of sustainability ((1) ecological, (2) social, (3) economic, and (4) institutional, as shown in Figure 2) that are essential to achieving sustainable life quality [85]. For designers to practice sustainability, they should include and assess these four pillars, from obtaining the resources to producing final products [86]. It is fundamental to incorporate constituents that adhere to consumption and production standards, including the use of the most appropriate technology, materials, and production processes to achieve zero carbon emissions and minimal non-renewable resource use, whilst paying attention to the impacts on human well-being [87].

**Figure 3:**
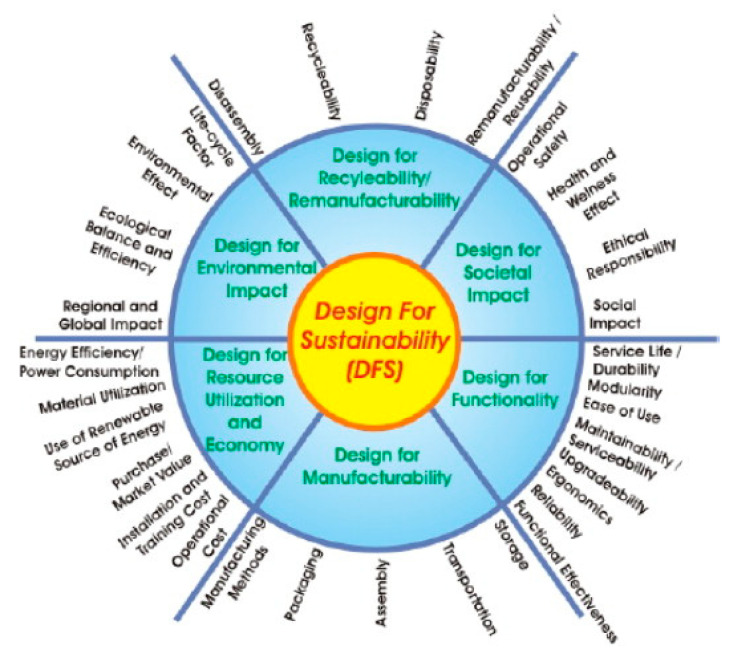
Elements within design for sustainability (DfS) [89].

Commonly, the DfS approach exploits the design for excellent (DfX) to produce a sustainable product. This process involves the analysis of the environmental impacts of specific design attributes, comprising safety and biodegradability prospects, in the development of sustainable components/products. Jawahir et al. [89] proposed a conceptual framework for DfS based on the DfX principles as displayed in Figure 3. The implementation of concurrent engineering in product development is essential to satisfying human needs and developing sustainable products before the manufacturing process [90]. In particular, a biocomposite product has to meet the requirements of life cycle analysis and sales trends, e.g., raw material and production costs, product’s performance, and consumer’s demands [91,92].

#### 4.3.1. Theory of Inventive Problem Solving (TRIZ)

**Figure 4:**
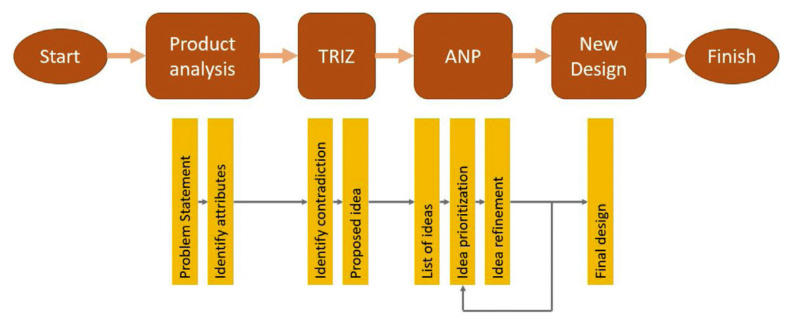
The application of TRIZ in the concurrent engineering conceptual design framework to develop the product [100].

TRIZ, or the theory of inventive problem solving, is a tool used by concurrent engineers to develop various solutions using inventive principles to cater to the problems that arise [93]. The tool also eliminates any negative drawbacks that may arise during the development of the solution, as it is focused on the root cause of the problem [94,95,96]. Initially, the tool is applied to determine the design intentions (purpose subject of the design) before the development of inventive solutions, specifically for the design. The TRIZ tool can be categorized into four main techniques: (1) Su-field modeling, (2) algorithms of inventive problem solving (ARIZ), (3) prediction of technology trends, and (4) contradiction engineering with 40 inventive principles [97]. The application of the techniques depends on the complexity level of the targeted problems when attempting to systematically solve a problem by identifying opportunity and innovation techniques [98]. In this case, Cascini et al. [99] developed a new concept of sheet metal snips based on TRIZ contradiction methods, which compare the improving and worsening parameters to select suitable inventive principles. At the end of the product development process, they refined the design concepts via a CAD optimization tool. Figure 4 displays the conceptual design process conducted by Asyraf et al. [100].

#### 4.3.2. Voice of Customer

Voice of customer (VOC) is one of the main approaches in concurrent engineering to generate ideas for design intends. The voice of the customer is obtained via many techniques, including direct customer specifications, observation, surveys, discussion or interviews, focus groups, warranty data, and field reports. From these VOC data, this information is then incorporated in a product planning matrix or quality function deployment (QFD) [101]. The QFD is used to define customer requests and turn this information into systematic plans to produce products to meet those desires [101].

#### 4.3.3. Morphological Chart

**Figure 5:**
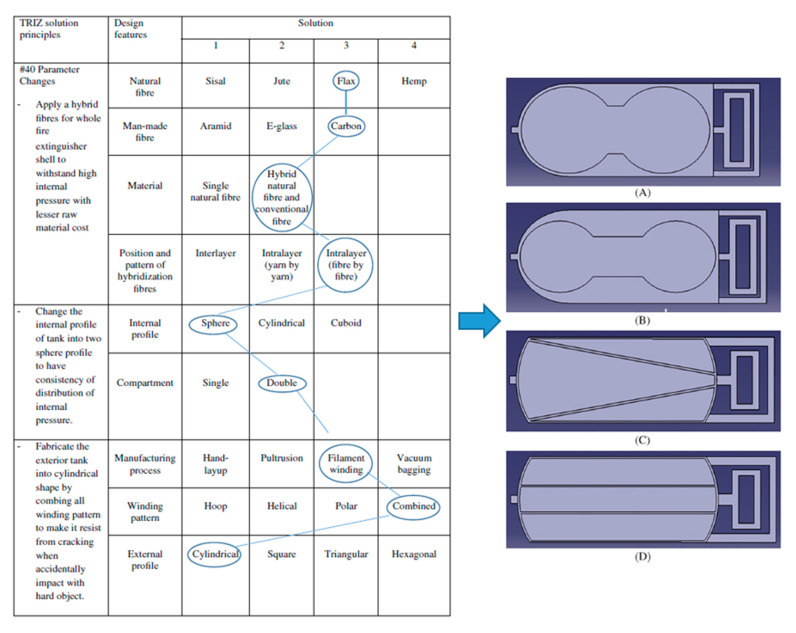
Morphological chart used to elaborate the design characteristics of a fire extinguisher, by Asyraf et al. [104]. (**A**)—concept design A, (**B**)—concept design B, (**C**)—concept design C, (**D**)—concept design D.

The morphological chart is a concurrent engineering technique that implements a chart with various arrangements to aid designers in selecting new combinations of attributes/elements. The “morphology” term refers to the study of the form or shape of the material, whereas “morphological chart” is defined as a systematic approach to generating and analyzing the form or characteristics of a product that might be selected [102,103]. The chart functions to offer a series of choices for each element and component that can be combined to become a solution idea. The combination of elements and components would create multiple design features beneficial for the product’s functions. Figure 5 displays an example of a morphological chart used to develop and model conceptual designs for a natural fiber composite fire extinguisher.

#### 4.3.4. Extending the Search Space

This concurrent engineering approach is also called the “Why? Why? Why?” technique, which is used to elaborate the search option by questioning the root cause of the problem [105]. For this case, the question would be “Why do we need safety in composite products?” After getting the answer to the question, it would be followed with another “why” question, until a conclusive solution is reached. For this case, the method is highly dependent on luck, thus brainstorming is suggested to solve the problems of the product’s development.

#### 4.3.5. Gallery Method

This technique is used by designers to produce ideas by displaying many generated concepts simultaneously with a conducted discussion. Typically, these concepts are visualized by sketching and taping them on the wall of the designer’s design room to review each aspect of the idea. Designers may consequentially be able to suggest improvements for the concept, or they might even suddenly generate related ideas via this process [106].

#### 4.3.6. Brainstorming

Analysis of the current systems or products is one of the methods used by many designers and researchers to initiate new models or prototypes with better solutions [107]. This type of method is also called brainstorming, which involves discussions covering the physical analysis of current products. The discussion might produce a clearer picture by mind mapping problems, generating ideas, producing concept designs, and determining fabrication processes and finalized prototypes [91]. Usually, the analysis of the existing product would be in relation to competitors’ products, older products of one’s own company, and similar products that have several sub-functions of function structures.

According to Sapuan et al. [108], several generation techniques have been used by designers to develop conceptual designs for products, e.g., theory of inventive problem solving (TRIZ), brainstorming, strengths, weaknesses, opportunities and threats (SWOT) analysis, gallery method, and systematic exploitation of proven ideas of experience [109,110]. For this project, the simplest way to generate the idea for conceptualizing the design concepts was using the brainstorming approach. A design focus group was formed for discussion among the members of Advanced Engineering Materials and Composites Research Centre (AEMC), Department Mechanical and Manufacturing Engineering, Universiti Putra Malaysia. Every concept design was outlined and listed based on discussion outputs and PDS documents of the previous design stage. In the end, around five conceptual designs of a roselle fiber-reinforced polymer composite smartphone holder were developed. The details of each concept design are laid out in Table 5.

In another study conducted by Ilyas et al. [111], a focus group was formed to comprehensively discuss and produce ideas on the conceptual design of a biocomposite mug pad among members of the Advanced Engineering Materials and Biocomposites Research Centre (AEMC), Department of Mechanical and Manufacturing Engineering, Universiti Putra Malaysia. After the brainstorming output, every concept design was listed based on the previous PDS document. Specifically, for this research activity, five design concepts of a roselle fiber biocomposite mug pad with details were produced and are tabulated in Table 6. Creative and innovative variations were developed to add value to the ideas, in addition to using roselle biocomposites.

## 5. Natural Fibers Composite Applications

**Figure 6:**
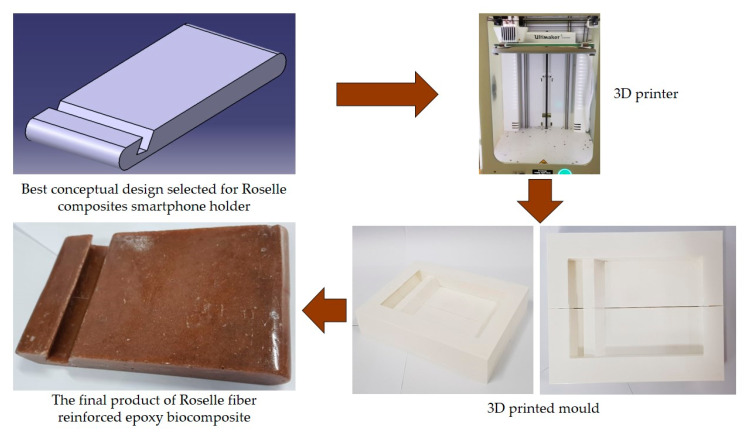
Smartphone holder using roselle fiber-reinforced polymer composites.

The applications for NFCs are growing rapidly in numerous engineering fields. Various types of natural fibers have been used as reinforcements in polymer composites, including corn [112], water hyacinth [113], coir [114], ginger [115,116], cotton [117,118], kenaf [7,91,119,120,121,122], sugarcane [123,124,125], flax [126], ramie [60], hemp [127], kapok [128], sisal [129], wood [22], oil palm [130,131], banana [132], as well as sugar palm [43,63,133,134,135,136,137,138,139,140,141]. Along with biodegradability, natural fibers come with many other advantages, e.g., substituting timber for wood plastic composite, being less costly, availability, and reducing deforestation [21]. Natural fibers have huge potential to be converted into useful products [20], as it was revealed by Ilyas et al. [142] that natural fibers are the right material for the replacement of glass and carbon fibers. Different natural fibers, e.g., jute, hemp, kenaf, oil palm, and bamboo-reinforced polymer composites, have become of great importance in different automotive applications, structural components, packaging applications, furniture, and constructions [46,143]. NFCs are used in electrical and electronic industries, aerospace, sports, recreation equipment, boats, machinery, office products, and so forth. A roselle fiber-reinforced polymer composite smartphone holder developed using the design for sustainability (DfS) approach was achieved by Sapuan et al. [108], as shown in Figure 6. The concept development of the environmentally friendly smartphone holder product was carried out using concept generation and concept evaluation techniques. The roselle composite smartphone holder development process involved market analysis, product design specification (PDS) document generation, conceptual design creation, and detailed design of the finished product. The mold of the product was fabricated using a 3D printing method. Then, the roselle fiber composite smartphone holder was fabricated via a hand lay-up process.

**Figure 7:**
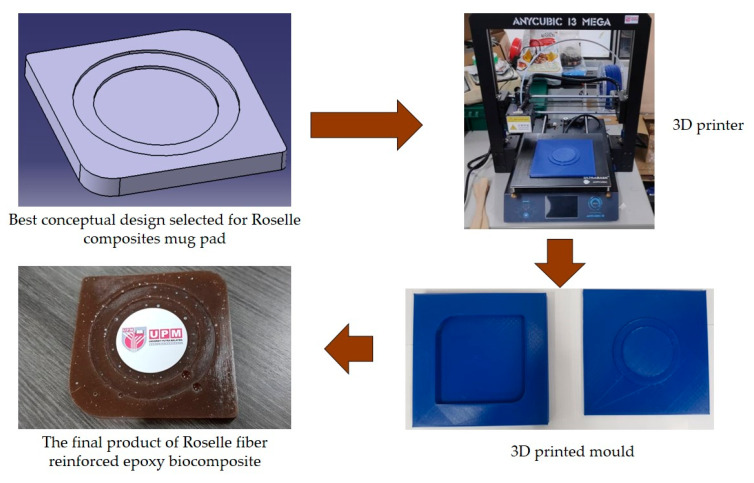
Mug pad holder using roselle fiber-reinforced polymer composites.

Another study was conducted by Ilyas et al. [111] on a roselle fiber-reinforced polymer composite mug pad’s product development process using the sustainability (DfS) approach, as shown in Figure 7. The concept development of the environmentally friendly mug pad product was performed using concept generation and concept evaluation techniques. The processes involved in their study were similar to those in the study of Sapuan et al. [108], in which the final design of the molded product was fabricated using a 3D printer, and the roselle fiber composite mug pad was fabricated using a hand lay-up process. The final product was completed and demonstrated easy fabrication, light weight, low overall cost, and an appropriate balance between functionality and aesthetics. Table 7 shows the example applications of the natural fiber composite.

### 5.1. Natural Fibers Composites’ Applications in Electrical and Electronic Components

**Figure 8:**
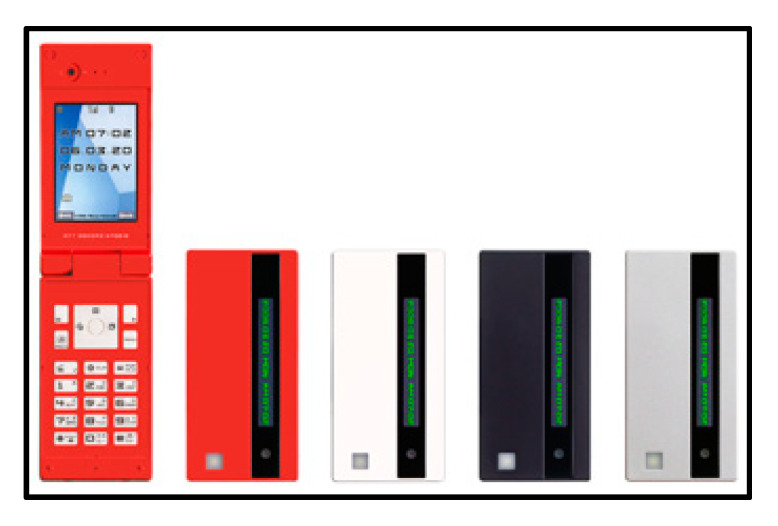
First mobile phone using a casing made of natural fiber material.

Currently, the increased importance of raw materials derived from renewable resources, as well as the recyclability or biodegradability of products, are causing a transformation from petroleum-based synthetics to natural fibers in electrical and electronic applications [31]. The broad advantages of natural fiber-reinforced composites, such as high stiffness to weight ratio, light weight, and biodegradability, make them suitable for different applications in electrical and electronic industries. “FOMA(R) N701iECO” utilize kenaf fibers in their eco-mobile phone casing, as shown in Figure 8.

### 5.2. Natural Fibers Composites’ Applications in Packaging

**Figure 9:**
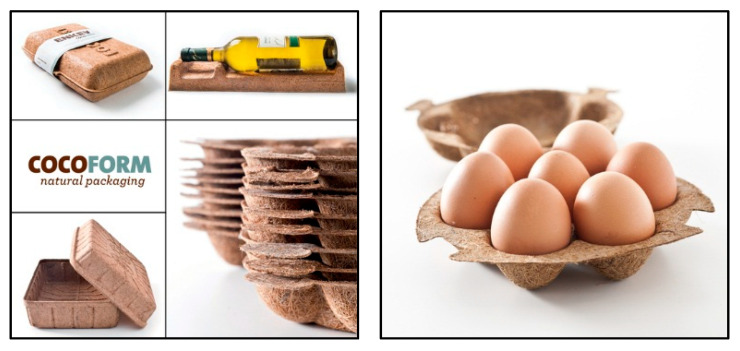
Packagings produced by Enkev Manufacturer from coconut fiber.

More recently, natural fiber composites have provided an alternative solution for better packaging. Previously, most of the petroleum-based plastics being used for food packaging have been non-degradable, causing many environmental problems associated with their disposal, including damage to the environment and eco-systems, water supplies, sewer systems, rivers and streams [144]. Moreover, they are non-renewable, and their prices are rising and unstable, given the impending depletion of petroleum resources. According to Ngo [145], the utilization of coir (coconut) fiber reinforced with natural latex, in place of synthetic materials, is of great interest for reducing the utilization of non-renewable and petroleum-based resources. Coconut fiber is a very tough yet also elastic material that hardly deteriorates at all over time. It is a durable material that can be re-used many times. After it is used, it can be recycled or disposed of without problem. After molding the material into the right shape, the material is heated to vulcanize the natural latex. The result is a very open structure that is strong and resilient. Figure 9 shows the packaging products produced by Enkev Manufacturer out of coconut fiber.

### 5.3. Natural Fibers Composites’ Applications in Sports Equipment

**Figure 10:**
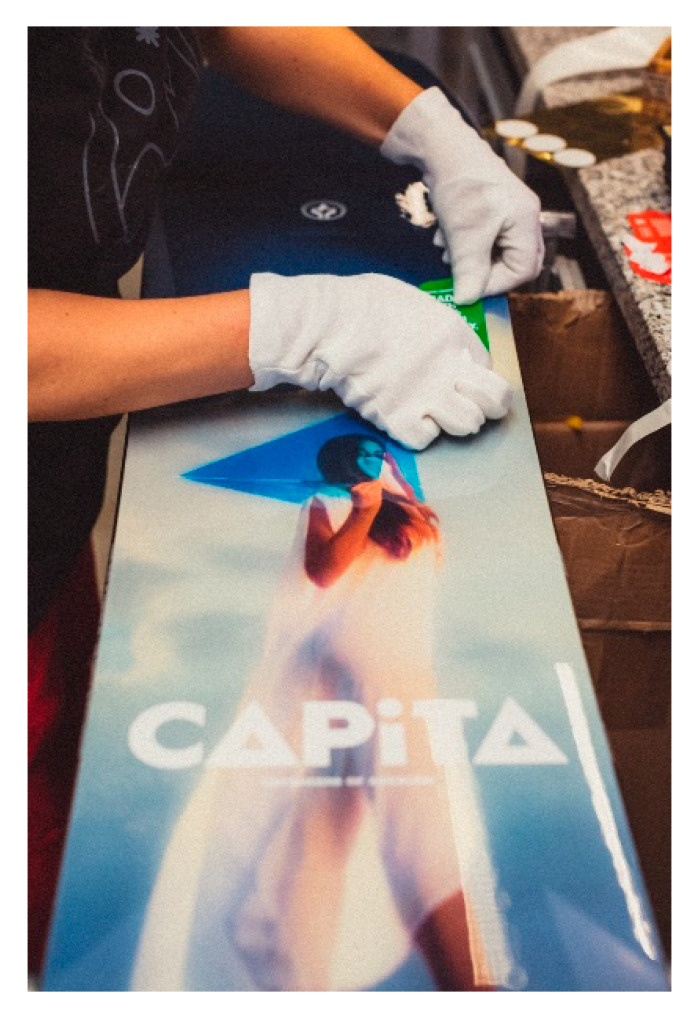
Bcomp Manufacturer’s longboards made from natural fiber.

Despite the most prominent applications of natural fiber composites being in the automotive industry, there are additional fields of application for natural fiber composites, such as in sports products. Before the advent of fiber-reinforced composites, sports equipment materials were made of wood, steel, stainless steel, aluminum, and alloy. In comparison with these materials, fiber reinforced composite materials have obvious advantages in the following aspects. The lower weight and relatively lower cost of natural fibers are the main aspects referred to as the reasons for the use of natural fiber composites in sports equipment. Most sports equipment relies on humans to move; therefore, lightweight equipment is desirable [146]. According to a study conducted by Yusup et al. [147], an oil palm empty fruit bunch fiber/epoxy composite that went through 24 h fiber treatment showed high potential to be used as a reinforcement to epoxy as a suitable material for sports equipment. Based on the results obtained related to the mechanical and physical properties, the composite of OPEFB fiber/epoxy had a flexural strength between 67.90 MPa and 83.63 MPa, which falls in the range of field hockey sticks’ strength requirements. The longboard shown in Figure 10 is one of the products made from AmpliTex^®^, bCores^®^, powerRibs, and natural fiber (flax, balsa wood) composite reinforcement materials [148].

## 6. Conclusions

This review article provides a compact and informative summary of natural fiber-reinforced polymer matrices from the perspective of product design development. Among the three main sources of natural fibers are plants, animals, and minerals, and these plant fibers or cellulosic fibers are in high demand, having developed since the resources they require are widely available, consume less energy, and are non-toxic to nature and humans. In general, natural fibers are made up of several main constituents, such as cellulose, hemicellulose, lignin, and pectin. Many researchers have discovered the good mechanical performance of these natural fibers due to the cellulose, which provides the good shape and structural integrity of the fibers. Thus, the integration of natural fibers with a polymer matrix in composites benefits various industries, as they exhibit low density, lower solidity, biodegradability, and cheapness compared to synthetic composites. Natural fiber composites are an effective way of improving the quality of products developed from them, in terms of environmental suitability, and economic and technical feasibility. The most common natural fibers used in composite products are flax, coir, hemp, and jute, while roselle, sugar palm, and kenaf are examples of emerging fibers due to their high mechanical strength and stiffness that are suitable for many engineering applications. It can be concluded that appropriate product design and manufacturing processes of NFPCs are required to enhance the properties of the products and their materials toward optimized strength and functionality. To ensure the optimization of the strength and functionality of natural fiber composite products, engineering design processes and techniques such as TRIZ, brainstorming, the voice of customers (VOCs), and morphological charts are essential. These techniques could define the problems of users and refine them in terms of the product’s functionality. In the end, an appropriate manufacturing process incorporates the product’s design and its applications. In the future, further research will be required to develop optimized engineering design techniques that complement the strength of the natural fiber composites, manufacturing processes, and functionality for heavy industry applications. Even now, natural fiber composites have the potential to be used in many applications that do not require very high load-bearing or high-temperature working capabilities.

## Figures and Tables

**Table 1:** Types of natural fibers [31,32,33,34].

**Natural Fiber**	Cellulose/Lignocellulose	Grass/Reed	Bamboo, corn
		Stalk	Wheat, maize, oat, rice
		Wood	Hardwood, softwood
		Fruit	Coir
		Seed	Cotton
		Leaf	Abaca, banana, pineapple, sisal
		Bast	Flax, hemp, jute, kenaf, ramie
	Animal	Wool/hair	Cashmere, goat hair, horse hair, lamb wool
		silk	Mulberry
	Mineral	-	Asbestos, ceramic, metal

**Table 2:** The advantages of natural fibers.

Author (Year)	Advantages of Natural Fibers
Bakar et al. [10]	Low cost, low elongation, low density, non-conductivity, corrosion resistance, absorb significant amounts and able to solve environmental pollution.
Corona et al. [35]	Renewable, moderate energy consumption for production and disposal can reduce environmental problems.
Hanan et al. [4]	Has certain strength properties, non-rough surface, lightweight, renewable, has specific modulus properties, can reduce pollution, biodegradable, require less energy to produce, and inexpensive.
Aji et al. [36]	Low density, cost-saving during manufacturing, less rough surface, harmless biodegradation, renewable, comparable mechanical properties with inorganic fiber, recyclable in most countries, and the surface is easily modified.
Amir et al. [37]	Substitute for synthetic fibers and as a reinforcing material in composites.
Nordin et al. [11]	In terms of mechanical properties, natural fibers are a good substitute for polymer composites because of their renewable material source, light weight, inexpensiveness, low density, and the materials are readily available.
Maleque et al. [38]	Ease of use in chemical and mechanical modifications.
Rognoli et al. [17]	Environmentally friendly materials.
Taekema and Karana, [39]	Low density, high specific strength, renewable, recyclable according to the mixture of materials used, high thermal and acoustic insulation, energy consumption savings of up to 60% in the production process (average for automotive component manufacturing), can be produced with low technology and investment and highly recommended for developing countries.
Sapuan and Maleque [40]	Mechanical properties are comparable to existing conventional materials that include low production costs, renewability, and environmentally friendly materials.
Shekar and Ramachandra [41]	Good mechanical properties, renewable, non-abrasive to process equipment, and can be burned at the end of its life cycle for energy recovery, and also abundantly available.
Elanchezhian et al. [9]	Renewable, inexpensive, completely or partially recyclable material, and biodegradable. In addition, this material has low density, low cost, and has environmentally friendly mechanical properties. It is also an alternative material for fiberglass, carbon, and human-made fibers for composite manufacturing.
Ilyas et al. [42,43,44]	Cost-effective, biodegradable, and renewable materials.
Peças et al. [31]	Renewable, low production costs, low density, acceptable modulus–weight ratio, low manufacturing energy consumption, low carbon, and biodegradable.
Huda et al. [45]	Cheaper, less energy required in the production of fiber reinforcement compared to conventional fibers such as glass and carbon.
Thyavihalli Girijappa et al. [46]	Abundantly available and cost-effective production.
Arpitha et al. [47]	Good mechanical properties, light weight, low cost, high specific strength, less rough surface, environmentally friendly, and good biodegradation characteristics.
Madhu et al. [48]	Creates huge employment opportunities in the rural plantation sector, available in large quantities, biodegradable, recyclable, better energy recovery, low production costs, lightweight materials, high strength and specific modulus, lower health risks, low density, low cost, less skin irritation, less abrasion of equipment, reduced tool wear, improved energy recovery, and reduced skin irritation and respiration

**Table 3:** Chemical composition of selected common natural fibers.

Fibers	Holocellulose (wt. %)		Lignin (wt. %)	Ash (wt. %)	Extractives (wt. %)	Crystallinity (%)	Ref.
	Cellulose (wt. %)	Hemicellulose (wt. %)					
Arecanut husk	34.18	20.83	31.60	2.34	-	37	[49]
Banana	7.5	74.9	7.9	0.01	9.6	15.0	[50]
Curauna	70.2 ± 0.7	18.3 ± 0.8	9.3 ± 0.9	-	-	64	[51]
Helicteres isora plant	71 ± 2.6	3.1 ± 0.5	21 ± 0.9	-	-	38	[52]
Kenaf bast	63.5 ± 0.5	17.6 ±1.4	12.7 ± 1.5	2.2 ± 0.8	4.0 ± 1.0	48.2	[53]
Kenaf core powder	80.26		23.58	-	-	48.1	[54]
Mengkuang leaves	37.3 ± 0.6	34.4 ± 0.2	24 ± 0.8		2.5 ± 0.02	55.1	[55]
Oil palm empty fruit bunch (OPEFB)	37.1 ± 4.4	39.9 ± 0.75	18.6 ± 1.3	-	3.1 ± 3.4	45.0	[56]
Oil palm empty fruit bunch (OPEFB)	40 ±2	23 ±2	21 ± 1	-	2.0 ± 0.2	40	[57]
Oil palm frond (OPF)	45.0 ± 0.6	32.0 ± 1.4	16.9 ± 0.4	-	2.3 ± 1.0	54.5	[56]
Oil palm mesocarp fiber (OPMF)	28.2 ± 0.8	32.7 ± 4.8	32.4 ± 4.0	-	6.5 ± 0.1	34.3	[56]
Phoenix dactylifera palm leaflet	33.5	26.0	27.0	6.5	-	50	[58]
Phoenix dactylifera palm rachis	44.0	28.0	14.0	2.5	-	55	[58]
Pineapple leaf	81.27 ± 2.45	12.31 ± 1.35	3.46 ± 0.58	-	-	35.97	[59]
Ramie	69.83	9.63	3.98	-	-	55.48	[60]
Rubber wood	45 ±3	20 ± 2	29 ± 2	-	2.5 ± 0.5	46	[57]
Soy hull	56.4 ± 0.92	12.5 ± 0.72	18.0 ± 2.5	-	-	59.8	[61]
Sugar beet	44.95 ± 0.09	25.40 ± 2.06	11.23 ± 1.66	17.67 ± 1.54	-	35.67	[62]
Sugar palm	43.88	7.24	33.24	1.01	2.73	55.8	[63]
Sugarcane bagasse	43.6	27.7	27.7	-	-	76	[64]
Water hyacinth	42.8	20.6	4.1	-	-	59.56	[65]
Wheat straw	43.2 ± 0.15	34.1 ± 1.2	22.0 ± 3.1	-	-	57.5	[66]

**Table 4:** Product design specification (PDS).

Design Specifications	Explanation
Universal design	Usable by both genders; availability of different sizes; usable by pilgrims with a beard, other facial hair, or other conditions that prevent a good seal between the face and the sealing surface of the face mask
Comfortable	Ergonomic; large breathing space (or dead space) for relaxed breathing; reduced facial covering without compromising the face mask’s efficiency
Effectiveness	Therapeutic effectiveness of the face masks against airborne infectious diseases is highly critical
Low cost	The low cost can allow face masks to be given for free by Tabung Haji, as preferred by the pilgrims

**Table 5:** New conceptual designs of a roselle fiber-reinforced polymer composite smartphone holder and their descriptions [108].

Conceptual Design		Description
1.	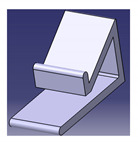	Concept inspired by high heel shoes. Lifts the smartphone higher than regular holder. Good artistic structure.
2.	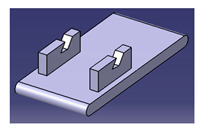	Easy to manufacture and assemble. Simple and minimalist design. High stability due to wider baseline.
3.	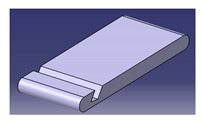	Simple and minimalist design. Easy for storage. Easy to fabricate and manufacture. High stability due to wider baseline.
4.	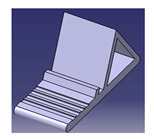	A hollow triangle feature can be used to hold stationary (i.e., pen, pencil, key, etc.). Three strip lines for aesthetics purposes.
5.	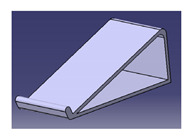	A hollow triangle feature can be used to hold stationery (i.e., pen, pencil, key, etc.). Simple and minimal design. Isometric design, hence easy to fabricate.

**Table 6:** Proposed conceptual designs of a roselle fiber biocomposite mug pad [111].

Conceptual Design		Description
1.	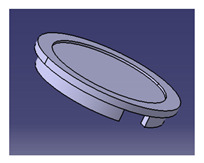	Concept inspired by the bottle cap. Composes of a main pad and two stand legs. Has a good artistic design.
2.	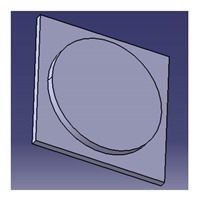	Comprises a bottom rectangle plate and a circle shape pad on the top. Simple and minimalist design. High stability due to a wider baseline.
3.	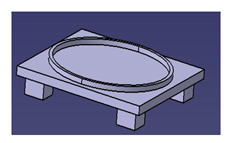	Concept inspired by a square coffee table. Composed of four legs, one ring to hold the mug, and one square pad. Complex shape with artistic value.
4.	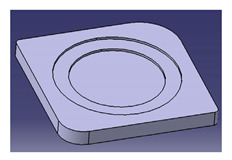	Simple and minimalist design. Easy for storage. Easy to fabricate and manufacture. High stability. High strength due to wider baseline. Concept inspired by a leaf.
5.	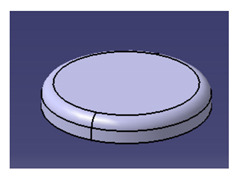	Simple and minimalist design. Circular and isometric design, hence easy to fabricate. Easy for storage. Easy to fabricate and manufacture. High stability.

**Table 7:** Applications of natural fibers composites [143].

Natural Fiber Composite	Applications
Bamboo	Application in building, construction, and others
Roselle	Mug pad, smartphone holder, furniture, automotive applications
Hemp	Construction products, textile, cordage, geotextile, paper and packaging, furniture, electrical, banknote, and pipe
Oil palm	Building materials such as window, door frame, structural insulated panel building system, siding, fencing, roofing, decking, and others
Wood	Window frame, panel, door shutter, decking, railing system, and fencing
Flax	Window frame, panel, decking, railing system, fencing, tennis racket, bicycle frame, fork, seat post, snowboarding, and laptop case
Rice husk	Building materials such as building panel, brick, window frame, panel, decking, railing system, and fencing
Bagasse	Window frame, panel, decking, railing systems, and fencing
Sisal	Used in the construction industry such as in panels, doors, shutting plates, and roofing sheet; also, in the manufacturing of paper and pulp
Stalk	Building panel, furniture panel, brick, drain, and pipeline
Kenaf	Packing material, mobile case, bag, insulation, clothing-grade cloth, soilless potting mix, animal bedding, and material that absorbs oil and liquids
Cotton	Furniture industry, textile and yarn, food packaging, and cordage
Coir	Building panel, flush door shutter, roofing sheet, storage tank, packing material, helmet and postbox, mirror casing, paperweights, projector cover, voltage stabilizer cover, filling material for seat upholstery, brush and broom, rope and yarn for net, bag, and mat, as well as padding for mattress and seat cushion
Ramie	Industrial sewing thread, packing material, fishing net, and filter cloth. It is also made into fabrics for household furnishings (upholstery, canvas) and clothing, as well as paper manufacture
Jute	Building panel, roofing sheet, door frame, door shutter, transport, packaging, geotextiles, and chipboard

## Data Availability

Not applicable.
